# Dark blood late enhancement imaging

**DOI:** 10.1186/s12968-016-0297-3

**Published:** 2016-11-07

**Authors:** Peter Kellman, Hui Xue, Laura J. Olivieri, Russell R. Cross, Elena K. Grant, Marianna Fontana, Martin Ugander, James C. Moon, Michael S. Hansen

**Affiliations:** 1 National Heart, Lung, and Blood Institute, National Institutes of Health, DHHS, 10 Center Drive MSC-1061, Bethesda, MD 20892 USA; 2 Children’s National Medical Center, 111 Michigan Ave., N.W, Washington, DC 20010 USA; 3 National Amyloidosis Centre, University College London (UCL) Medical School, Royal Free Hospital, London, UK; 4 Department of Clinical Physiology, Karolinska Institutet and Karolinska University Hospital, Stockholm, Sweden; 5 Barts Heart Centre, St. Bartholomew’s Hospital, London, UK

**Keywords:** Late enhancement, Gadolinium, Myocardial infarction, Dark blood, Ablation, Scar, PSIR, MOCO, LGE

## Abstract

**Background:**

Bright blood late gadolinium enhancement (LGE) imaging typically achieves excellent contrast between infarcted and normal myocardium. However, the contrast between the myocardial infarction (MI) and the blood pool is frequently suboptimal. A large fraction of infarctions caused by coronary artery disease are sub-endocardial and thus adjacent to the blood pool. It is not infrequent that sub-endocardial MIs are difficult to detect or clearly delineate.

**Methods:**

In this present work, an inversion recovery (IR) T2 preparation was combined with single shot steady state free precession imaging and respiratory motion corrected averaging to achieve dark blood LGE images with good signal to noise ratio while maintaining the desired spatial and temporal resolution. In this manner, imaging was conducted free-breathing, which has benefits for image quality, patient comfort, and clinical workflow in both adults and children. Furthermore, by using a phase sensitive inversion recovery reconstruction the blood signal may be made darker than the myocardium (i.e., negative signal values) thereby providing contrast between the blood and both the MI and remote myocardium. In the proposed approach, a single T1-map scout was used to measure the myocardial and blood T1 using a MOdified Look-Locker Inversion recovery (MOLLI) protocol and all protocol parameters were automatically calculated from these values within the sequence thereby simplifying the user interface.

**Results:**

The contrast to noise ratio (CNR) between MI and remote myocardium was measured in *n* = 30 subjects with subendocardial MI using both bright blood and dark blood protocols. The CNR for the dark blood protocol had a 13 % loss compared to the bright blood protocol. The CNR between the MI and blood pool was positive for all dark blood cases, and was negative in 63 % of the bright blood cases. The conspicuity of subendocardial fibrosis and MI was greatly improved by dark blood (DB) PSIR as well as the delineation of the subendocardial border.

**Conclusions:**

Free-breathing, dark blood PSIR LGE imaging was demonstrated to improve the visualization of subendocardial MI and fibrosis in cases with low contrast with adjacent blood pool. The proposed method also improves visualization of thin walled fibrous structures such as atrial walls and valves, as well as papillary muscles.

## Background

Late gadolinium enhancement (LGE) has become a gold standard in myocardial viability assessment [1, 2] providing excellent depiction of myocardial infarction (MI) and macroscopic scarring. The use of late enhancement in the diagnosis of ischemic heart disease and in guiding therapy such as revascularization has gained wide acceptance. More recently, late enhancement is playing a broader role in characterizing fibrosis in non-ischemic cardiomyopathies [3, 4], and in measurement of scar resulting from treatment of cardiac arrhythmias using radiofrequency ablation [5]. As the use of late enhancement imaging has matured and as the span of applications has widened, clinicians are examining late enhancement images for more subtle indication of fibrosis and the demands on image quality have grown [6].

Late-enhancement imaging typically achieves excellent contrast between infarcted and normal myocardium. However, the contrast between the MI and the blood pool is frequently suboptimal. A large fraction of infarctions caused by coronary artery disease are sub-endocardial and thus adjacent to the blood pool. The contrast between the blood and MI in the inversion recovery (IR) image depends on variables such as contrast agent dosage, time from gadolinium administration, clearance rate, and imaging parameters. Blood velocity may also have a role in the contrast, even though non-slice-selective IR is used. Therefore, as a result of mechanisms that are not fully characterized or controlled, it is not infrequent that sub-endocardial MIs are difficult to detect or clearly delineate.

Imaging at a later time point can result in better blood pool contrast for some subjects, but it may not be practical from the stand point of clinical workflow to wait too long, and in some instances contrast may worsen. Technical solutions to this problem are to use multiple contrasts such as T1 and T2 [7, 8], or to use blood suppression techniques [9–15]. The T2 of blood (250 ms) is significantly longer than that of myocardium (45 ms) and may be used to discriminate the MI from blood pool. In the “Multi-contrast delayed enhancement” (MCODE) approach [6, 7, 16], both T1-and T2-weighted images are acquired within the same breath-hold. The T1-weighted image is a phase sensitive inversion recovery (PSIR) image, and the T2-weighted image uses an RF preparation for T2-weighting. Both images are acquired at the same diastolic cardiac phase and are spatially registered facilitating fusion of the images to enhance the sub-endocardial border. An alternative approach that also exploits the difference in tissue T1 and T2 is to use a segmented cine IR with steady state free precession (SSFP) readout [8]. In this method, each cardiac phase has a unique T1 and T2 contrast as determined by both the inversion recovery and the SSFP readout which has a $\sqrt{T2/T1}$ steady state dependence. The blood will appear dark at a different cardiac phase than the MI. In an alternative approach a IR fast low angle shot (FLASH) sequence may be modified to null both the blood and the normal myocardium using 2 inversions with carefully chosen inversion times [10] or using a triple inversion scheme [14].

Alternatively, LGE with blood suppression may be achieve my combining either a T2 preparation [9, 11, 13] with IR or by combining a magnetization transfer (MT) preparation with IR [12, 15]. In these schemes, the myocardial signal is reduced relative to the blood signal thereby reducing the inversion times to null the myocardium. In this way, it is possible to null both the myocardium and the blood at the same time. The order of the T2 and IR preparations may be applied as T2-IR [13] or IR-T2 [9]. Both of these previously reported schemes used a FLASH readout. In this present work, we combined a IR-T2 with a single shot SSFP readout and respiratory motion corrected averaging [6, 17, 18] to achieve the acceptable SNR while maintaining the desired spatial and temporal resolution. In this manner, imaging is conducted free-breathing which has benefits for image quality, patient comfort, and clinical workflow in both adults [19] and children [20]. Furthermore, by using a PSIR reconstruction [21] the blood signal may be made darker than the myocardium (i.e., negative signal values) thereby providing contrast between the blood and both the MI and remote myocardium.

In conventional bright blood LGE, the contrast-to-noise ratio for detection of scar is optimized by nulling the normal myocardium [22]. Selection of the inversion time (TI) may be performed manually, or aided by a IR-cine scout [23]. For dark blood (DB) LGE methods such as T2-IR, IR-T2, or MT-IR, there are more degrees of freedom and additional imaging protocol parameters must be specified to achieve the desired myocardial null and blood signal contrast. In the proposed approach, a single T1-map scout is used to measure the myocardial and blood T1 using a MOdified Look-Locker Inversion recovery (MOLLI) protocol [24] and all protocol parameters are automatically calculated from these values within the sequence thereby simplifying the user interface and avoiding the necessity of a lengthy scout sequence. The use of PSIR further avoids the requirement for precise nulling [6].

Dark blood LGE schemes provide contrast between the MI and the blood pool at the expense of SNR. The SNR cost of the proposed imaging method is characterized through simulation and verified experimentally. It is possible to recover lost SNR by means of respiratory motion corrected averaging with free breathing acquisition.

The proposed dark blood PSIR LGE is demonstrated for several applications. One application, which motivated the initial development, was peri-procedural cardiovascular MR in children undergoing radiofrequency catheter ablation for ventricular tachycardia (VT). The concern was the potential poor contrast with the adjacent blood pool for acute subendocardial lesions. Patients undergoing such procedures are anesthetized and all imaging should ideally be acquired under free-breathing conditions. Furthermore, imaging had to be performed rapidly. The dark blood method was also used for LGE on subjects with both ischemic and non-ischemic heart disease in both adult and paediatric populations.

## Methods

### Dark-blood PSIR LGE

**Fig. 1:**
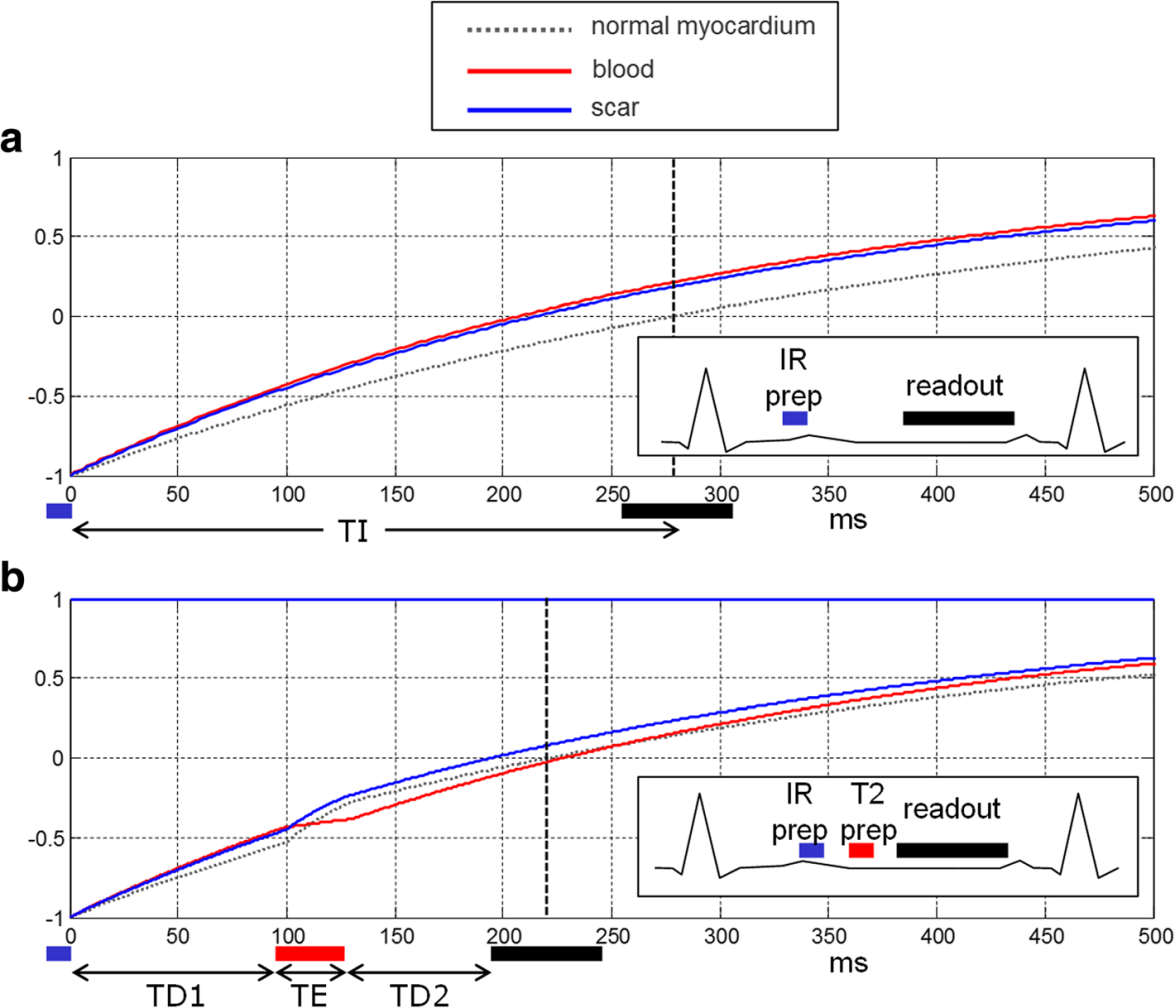
**a** inversion recovery for bright blood PSIR LGE in case with scar signal (*blue*) less than blood (*red*) resulting in poor contrast. **b** inversion recovery for Dark Blood (DB) PSIR using combined IR and T2 preparation to shift the null time of blood relative to the normal myocardium. In this case the delays are chosen such that the blood signal (*red*) is less than the myocardium (*dashed gray*) resulting in dark blood using PSIR reconstruction, which preserves the signal polarity. Inversion times to null the normal myocardium are depicted by vertical dashed lines. The loss in SNR due to the T2 preparation is mitigated by increased respiratory motion corrected averaging (MOCO)

Dark Blood (DB) LGE was implemented by adding a T2 prep between the IR preparation and the readout [9]. This shifts the null time of the myocardium relative to blood making it possible to choose delays that simultaneously null both myocardium and blood. Figure 1 illustrates the case where the intensity of the inversion recovery signal for the scar is less than the blood leading to poor contrast for conventional LGE (Fig. 1 (a)). By addition of a T2 preparation (Fig. 1 (b)) and judicious choice of delays a positive contrast between both the scar and normal myocardium with the blood is achieved. In this example, the choice of delay parameters (TD1, TE, and TD2) for the IR-T2 preparation are chosen to null the normal myocardium prior to the blood which makes the blood signal negative when reconstructed using a phase sensitive approach (PSIR), and thus will appear darker than the myocardium. Contrast between the normal myocardium and blood is important in order to delineate the myocardium wall.

With the IR-T2 approach to DB LGE, the myocardium signal is attenuated to achieve an earlier null time. Although blood suppression improves the contrast-to-noise ratio (CNR) between MI and blood, the myocardial signal attenuation corresponding to the T2 weighting exp (−TE/T2) causes a loss in CNR between the MI and remote normal myocardium as seen in Fig. 1. In the proposed method, free-breathing PSIR LGE imaging was performed using a single shot SSFP sequence with respiratory motion correction (MOCO) averaging of repeated measurements [6, 17, 18]. The number of averages was double for the dark blood protocol as compared to the bright blood PSIR MOCO protocol in order to improve the CNR by $\sqrt{2}$, and thereby recover some of the loss due to the T2 weighting.

The MOCO is done independently for the IR and proton density (PD) images and the MOCO averaged images are co-registered prior to the PSIR demodulation step. A non-rigid image registration is used for in-plane motion correction and PD/IR co-registration, and in-plane motion is dealt with by discarding 50 % of the acquired measurements which are most dissimilar. Non-rigid registration was performed pairwise between a target and reference image. For each pair, the algorithm estimates a deformation that maximizes the local cross-correlation between the reference and the uncorrected (target) image using a fast variational approach [25, 26] that can be considered as an extension of the classic optical flow method. The process is implemented in a multi-scale approach from coarse to fine resolution which increases the speed and provides improved convergence. Once the non-rigid motion (deformation) field is estimated, the images are warped using a sub-pixel spline based interpolator. The selection of the reference frame used for image registration as well as which frames to be discarded is based on the similarity of frames as estimated from a global mean square difference metric [17]. In this way, the retrospective image based strategy averages the most frequent respiratory phase which is typically at end-expiration. The image reconstruction pipeline was implemented and deployed using the Gadgetron image reconstruction framework [27].

**Fig. 2:**
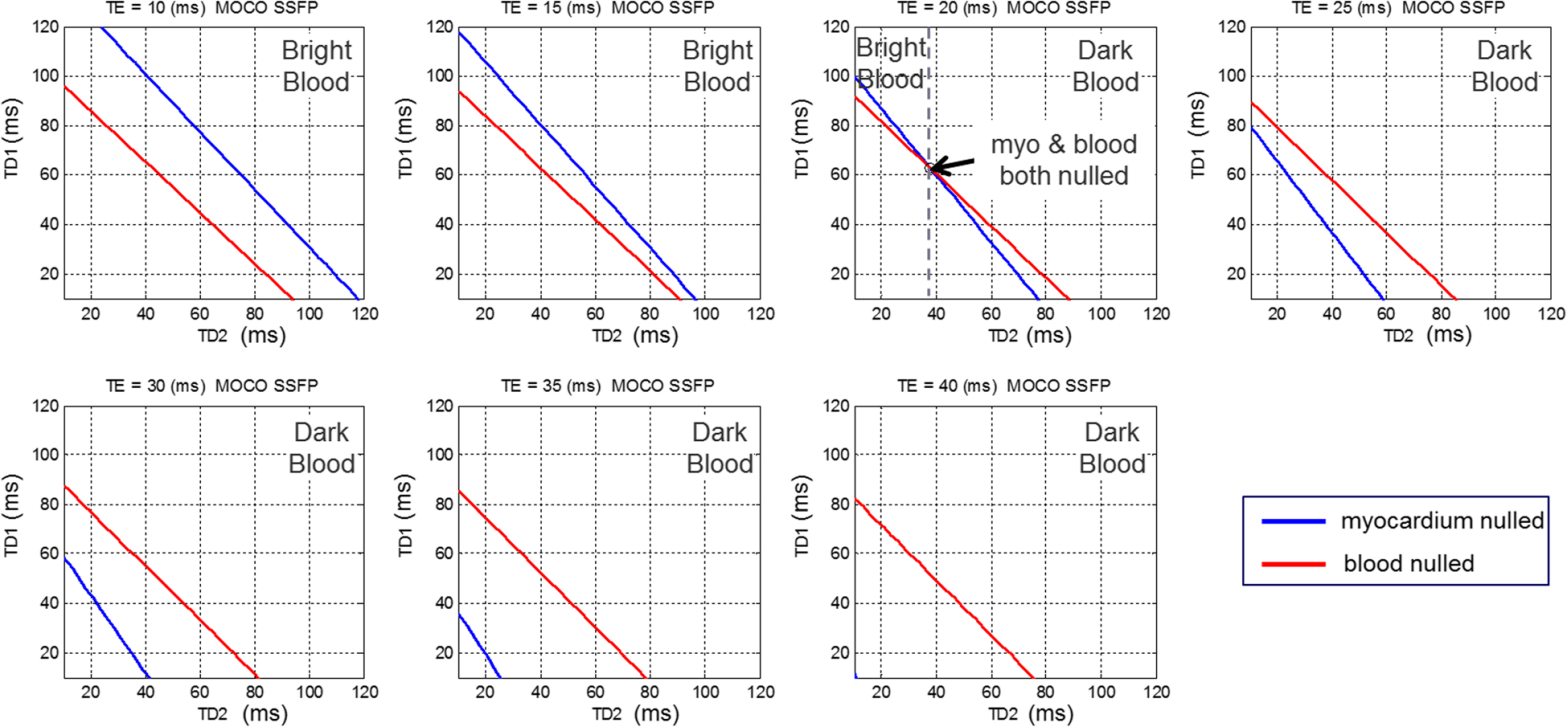
Calculation of delays TD1 and TD2 for various TE for specified myocardial and blood T1 of 500 ms and 350 ms respectively. TD1 and TD2 are calculated to null the normal myocardium (*point on blue line*) to achieve a specified blood suppression. In this case, dark blood may be achieved for TE ≥ 20 ms. The spacing between the *blue* (myocardial null) and *red* (blood null) indicated the degree of blood suppression with dark blood (blood < myocardium) when *red* line is above the *blue* line. Calculations are performed interactively on the scanner in response to protocol changes (TR, FA, matrix size, etc.)

Unlike the conventional PSIR LGE, which has a single inversion time parameter (TI) to specify in order to null the normal myocardium, the DB PSIR LGE has 3 parameters, TD1, TE, and TD2. In order to facilitate the selection these parameters, a complete Bloch simulation was performed using the complete imaging protocol, patient heart rate, measured T1 values of the remote normal myocardium and blood pool, and assuming nominal values for myocardial T2 (45 ms) and blood T2 (250 ms). The simulation determined the delay times TD1 and TD2 which null either the myocardium or blood for a given T2 prep echo time (TE) by calculating the magnetization for each readout of the inversion recovery signal. The calculated null times are shown in Fig. 2 for an example protocol (T1_myo_ = 500 ms, T1_blood_ = 350 ms) with null times for blood in red and myocardium in blue for a set of TE’s. For TE = 20 ms, the red and blue curves intersect at TD1 = 63 ms and TD2 = 38 ms. At this intersection, TD1 = 63/TD2 = 38/TE = 20, the normal myocardium and blood are simultaneously nulled. For this TE (20 ms) and for points on the blue line with TD2 > 38 ms, the myocardium is nulled and the blood will have negative values (i.e., not recovered past zero) and will appear dark with respect to the normal myocardium, and for TD < 38 the myocardium will have a positive signal. For TE ≥ 25 ms, the blood will be dark, in this example, and for TE <20 will appear bright. The spacing between the blue and red lines will govern the degree of blood suppression.

**Fig. 3:**
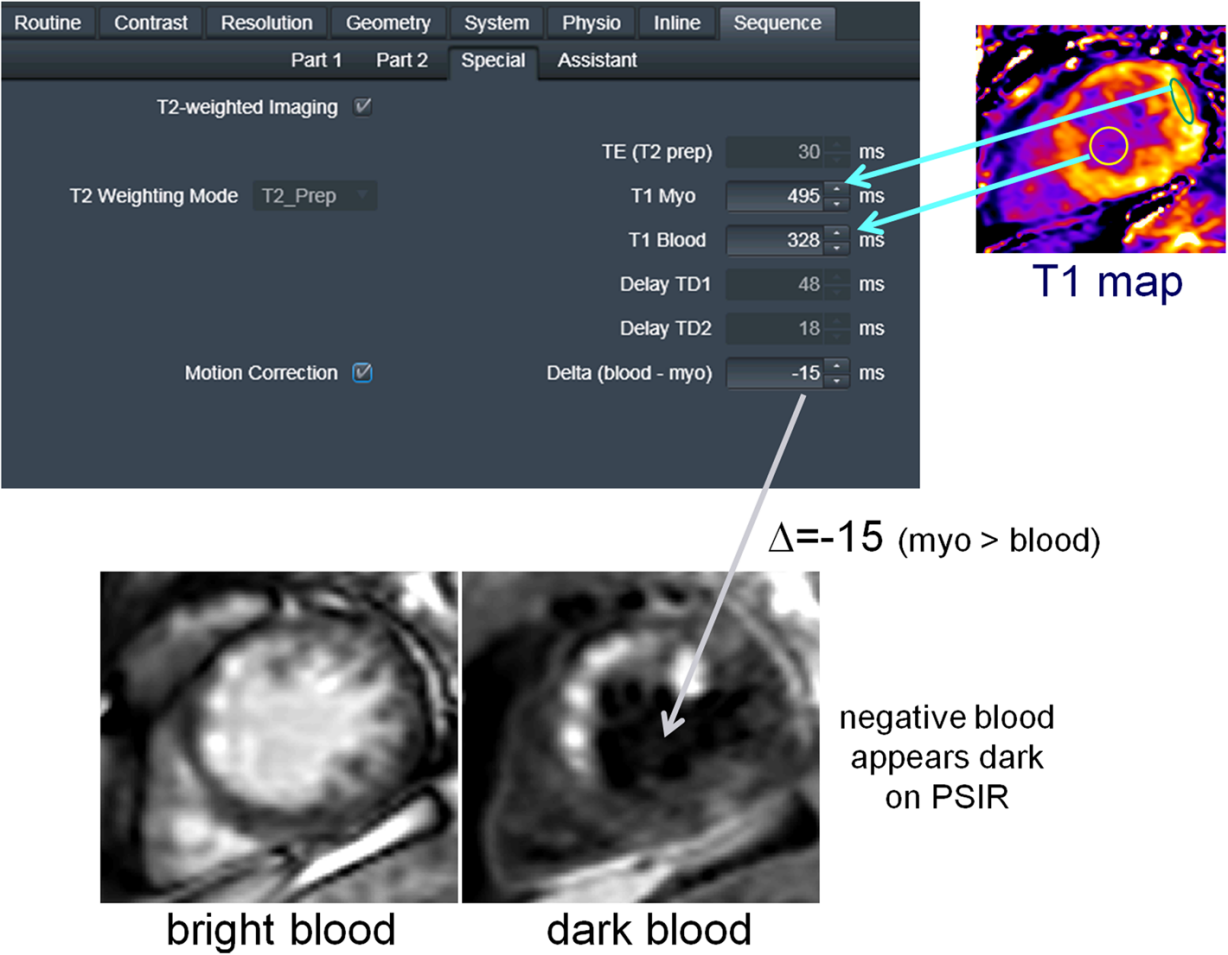
User Interface for Dark Blood (DB) Protocol: (1) User acquires T1 map and measures ROI values for remote blood and myocardium. (2) **a** enter ROI values in sequence user interface, **b** enter degree of blood suppression as “delta” value in user interface. (All other parameters (TE, TD1, TD2) are calculated automatically). (3) Acquire images free-breathing

Typical imaging parameters are listed in Table 1 for both the bright and dark blood PSIR MOCO protocols. The myocardial and blood T1 are measured using a T1-mapping scout scan using the MOdified Look-Locker Inversion recovery (MOLLI) approach [24] with a modified protocol using respiratory motion correction and a 4 s (1 s) 3 s (1 s) 2 s acquisition scheme [28] that is acquired in 11 s. The measured T1 values are entered on the scanner in the user interface (UI) (shown in Fig. 3). The blood suppression is specified as a value (delta) which is dark when the value delta < 0. The sequence calculates TD1, TD2, and TE using a strategy that seeks to achieve the desired blood suppression with the shortest TE so as to minimize SNR loss. In order to speed up the computation, a series of rotation matrices were pre-computed and stored as look-up-tables (LUT). In this way, the calculation was fast enough that the user interface was responsive to changes in imaging protocol parameters. The values for TD1, TD2, and TE are thereby updated on the fly when any protocol parameter is modified (e.g., matrix size, flip angle, etc.).

**Table 1:** Imaging protocol parameters

	Bright Blood (BB)	Dark Blood (DB)
Preparation	Inversion Preparation	Inversion Preparation & T2 preparation
Readout	Single shot, SSFP	
	(FA_IR_ = 50°, FA _PD_ = 8°)	
Typical FOV/resolution	360 × 270 mm^2^	
	1.4 × 1.9 × 8 mm^3^	
Matrix size	256 × 144 (parallel imaging factor 2)	
Number of acquired measurements	8	16
T2 prep TE	10–40 ms	
TE/TR	1.2/2.8 ms	
ECG triggering	Inversions every 2 RR (HR < 90 bpm)	
	Inversions every 3 RR (HR > 90 bpm)

The Bloch simulation used to generate the LUTs included a waveform level calculation of the adiabatic T2 prep and hypersecant inversion pulses as well as shape of RF excitation pulses, which determine the slice profile. The T2 prep was a custom design 0° BIR4 with single refocusing pulse capable of achieving TE as short as 10 ms.

### In-vivo studies

In-vivo studies were performed to validate the concept, measure the performance, and evaluate the user interface in a clinical workflow. To meet this objective, a pilot study was performed at 4 sites: Children’s National Medical Center (CNMC), Washington, D.C., Bart’s Heart Centre, St. Barthomolew’s Hospital, London, UK, The National Amyloidosis Centre, UCL Royal Free Hospital, London, UK, and Karolinska University Hospital, Stockholm, Sweden. Studies were approved by the local Ethics Committees and Internal Review Boards at each institution. Data was acquired for 61 subjects (Bart’s 39, Karolinska 9, Royal Free 9, CNMC 4). Initial data was used to optimize the protocol parameters (number of averages and blood suppression “delta” parameter). After initial protocol optimization, raw data was saved to facilitate SNR measurement. CNR measurements were made on 30 subjects (Bart’s 21, Karolinska 5, Royal Free 4) with clearly identifiable MI. Anonymized data was analysed at NIH with approval by the NIH Office of Human Subjects Research OHSR (Exemption #13156). All imaging was performed at 1.5 T (Magnetom AERA, Siemens, software version VE11A). Gadolinium (Gd) contrast agent (Gadobutrol or Gadoterate meglumine) and dosage (0.1-0.2 mmol/kg) varied between sites. LGE images were acquired between 10–20 min following administration of Gd contrast. In this initial technical development study, both dark blood and bright blood PSIR LGE MOCO imaging was performed at the end of the study for all consented subjects for which the additional time could be accommodated in the clinical workflow at the discretion of the radiographer performing the scan. At the completion of bright blood PSIR imaging the dark blood imaging was performed for a single slice, followed by a repeated bright blood PSIR MOCO LGE. Both the dark blood and bright blood images analysed were acquired within 1 min. The acquisition duration for the bright blood approach was 16 heart beats per slice (8 measurements × 2 RR) and for the dark blood approach was 32 heart beats (16 measurements).

### CNR Measurements

The contrast-to-noise ratios (CNR) of interest are the difference of SNR between MI and remote (CNR_MI-remote_), between MI and blood pool (CNR_MI-blood_), and between blood and remote (CNR_blood-remote_). CNR measurements were made by subtracting the SNR from manually drawn regions of interest on SNR scaled image reconstructions [29, 30]. SNR scaled uniform noise, PSIR MOCO average images prior to surface coil correction were used. This method accounts for all factors including the parallel imaging losses due to so-called g-factor. Measurements were made on *N* = 30 studies for which there was positive identification of scar.

## Results

### CNR measurements

**Fig. 4:**
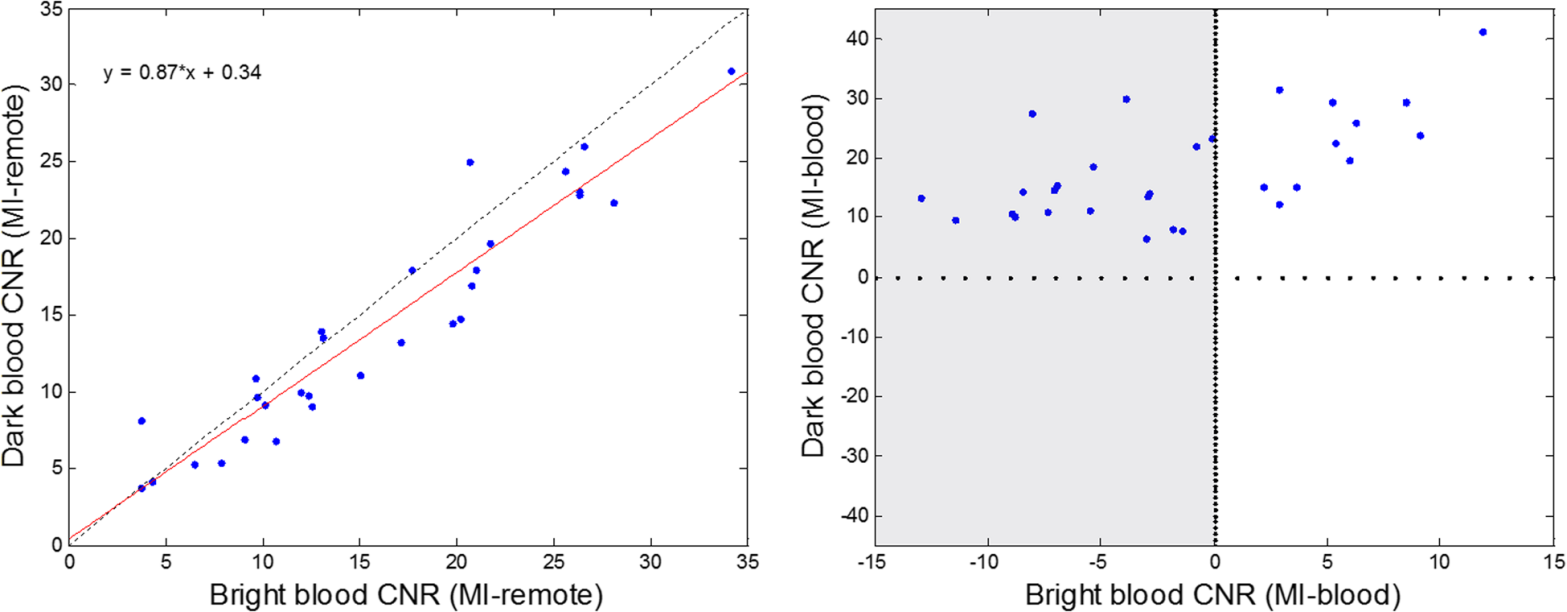
CNR measurements between MI and remote (left) and MI and blood pool (right) for *N* = 30 patients. Note that using the dark blood PSIR LGE the contrast between the MI and blood (CNR_MI-blood_) is positive for all cases, whereas a large number of conventional bright blood PSIR have poor contrast between the MI and blood (CNR_MI-blood_ < 0) as region shaded in *gray*

The CNR_MI-remote_ for dark blood (DB) and bright blood (BB) protocols in Table 1 were measured for *N* = 30 patients (Fig. 4). The CNR between MI and remote myocardium using the 2 protocols had a linear fit DB-CNR_MI-remote_ = 0.87 BB-CNR_MI-remote_ + 0.38 corresponding to a 13 % loss in CNR for the dark blood protocol (95 % confidence of slope was 0.76 to 0.98). Using the dark blood PSIR LGE protocol, the contrast between the MI and blood (CNR_MI-blood_) was positive for all cases, whereas a significant fraction (63 %) of the conventional bright blood PSIR cases have poor contrast between the MI and blood (CNR_MI-blood_ < 0) as region shaded in gray. The CNR between the blood and remote myocardium was 17.4 ± 7.1 (m ± SD) for the bright blood protocol, and−3.9 ± 3.2 for the dark blood protocol.

### Example studies

**Fig. 5:**
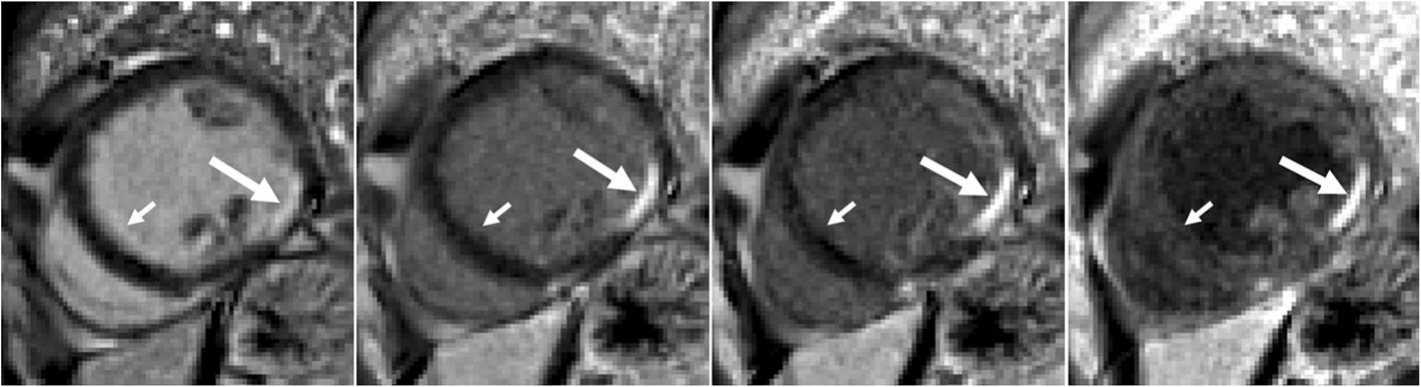
Bright blood PSIR LGE (left) and dark blood PSIR LGE with varying blood suppression. The subendocardial MI (*long arrow*) has increasing contrast with the blood pool as the blood suppression is increased. Gibb’s ringing (*short arrow*) of LV blood pool at septal border in bright blood LGE (left) is not present in DB LGE

**Fig. 6:**
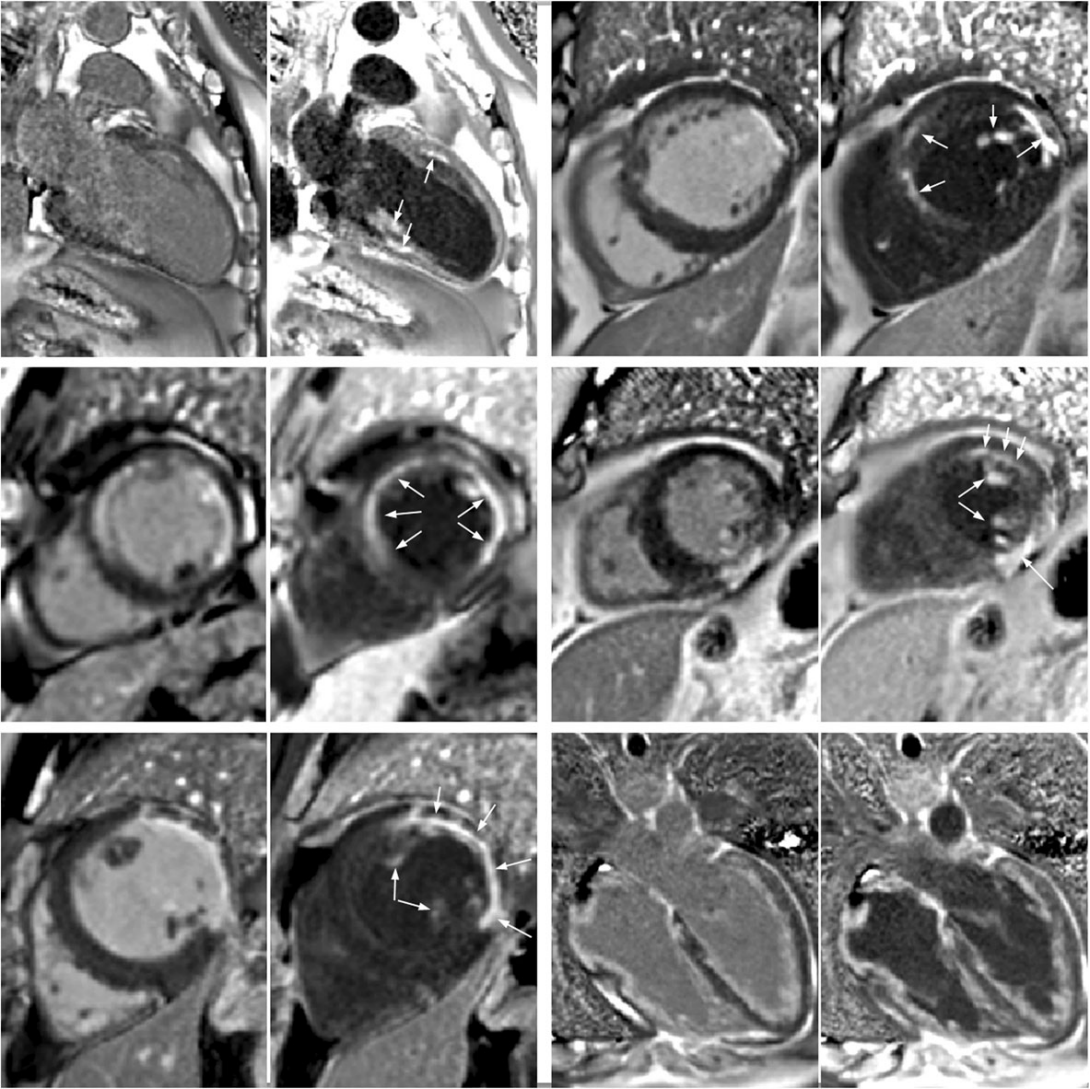
Example of bright and dark blood PSIR LGE for 6 adult patient studies with subendocardial LGE

**Fig. 7:**
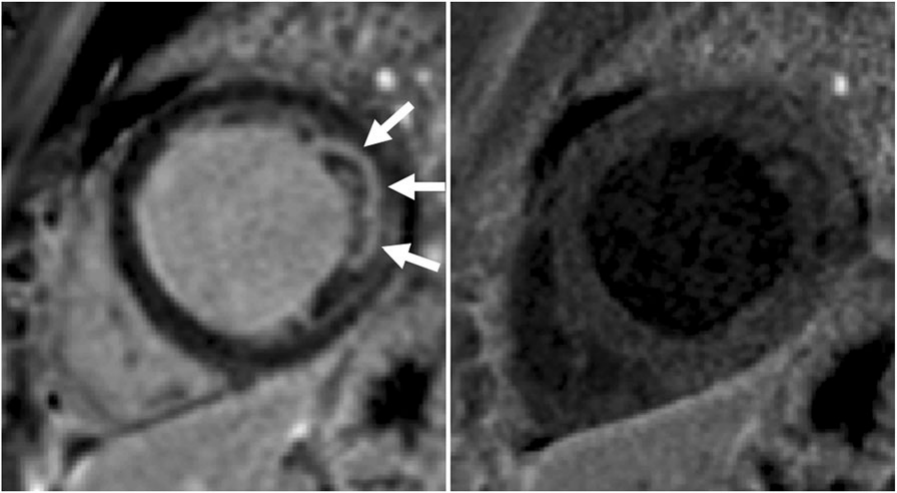
Example of dark blood PSIR LGE (right) showing that a possible lateral infarct on the bright blood (left) is in fact just blood pool signal in the trabeculae

**Fig. 8:**
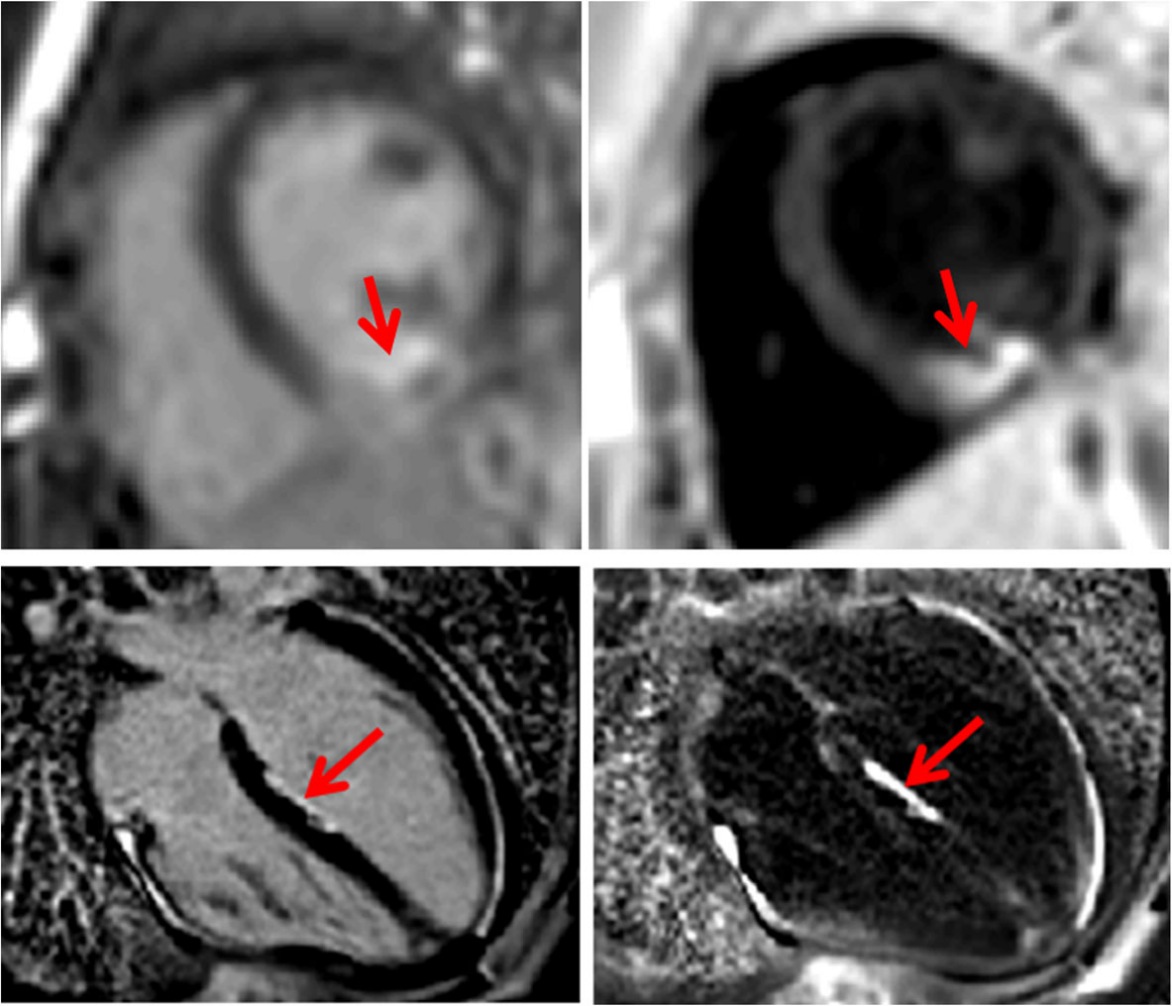
Free-breathing PSIR MOCO LGE acquired immediately following ablation in subject 1 under sedation (2 year old male) using bright blood (left) and dark blood (right) protocols shows acute ablation lesion (top row) and subject 2 (14 year old female) in 2-week follow-up study with ablation lesion in LV septum (bottom row)

**Fig. 9:**
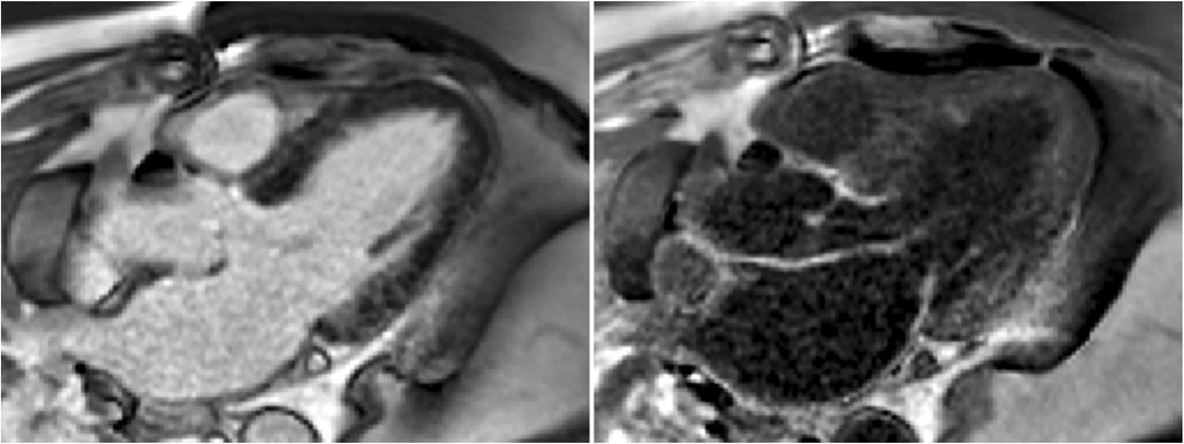
PSIR LGE images in 3 chamber view illustrating the improved visualization of thin walled atria and valve structures using DB (right). Note that the thin walled structures have greater enhancement than the myocardium due to fibrous composition leading to higher gadolinium concentration with shorter T1 than myocardium

The degree of blood suppression may be varied by adjusting the user specified “delta” parameter on the sequence user interface (Fig. 5). The “delta” parameter was +20, +5, and−15 ms (from left to right) for the 3 blood suppressed images. The inferior MI (long arrows) is most clearly defined with the blood suppression set for dark blood (right) which is used by default. Slight Gibb’s ringing of the LV blood pool observed in the septal region (short arrows) for the bright blood PSIR LGE image which may mimic LGE is clearly not evident in the blood suppressed images. Examples of PSIR LGE positive cases by both bright and dark blood methods are shown in Fig. 6 for a series of studies. The areas of scar indicated by LGE are more conspicuous on the DB PSIR images (see arrows) and in some cases might have been missed entirely in the bright blood PSIR images. Although an MI is clearly present in Fig. 6 middle row right, multi-vessel involvement seen in the DB PSIR image is difficult to assess in the bright blood image. LGE in papillary muscles has positive contrast in the DB PSIR. In the case with amyloidosis (Fig. 6 lower right), subendocardial fibrosis appears more distinct in the DB PSIR image, particularly in LV. In Fig. 7 is an example of a case (7 year old male with dilated cardiomyopathy) in which the dark blood PSIR LGE (right) suppressed a bright signal in the myocardium on the bright blood PSIR LGE, which could have been erroneously classified as a lesion, but turned out to be due to blood trapped in trabeculae. There is also Gibb’s ringing of the LV blood pool in the septum region for the bright blood LGE image. The PSIR LGE images in Fig. 8 are from a VT ablation case. Free-breathing PSIR MOCO LGE was acquired immediately following ablation in a 2 year old male (top row) and at a 2 week follow-up study for a 14 year old female (bottom row). Figure 9 illustrates the improvement in visualization of thin wall structures such as atrial walls and valves.

## Discussion

The conspicuity of subendocardial fibrosis and MI is greatly improved by dark blood (DB) PSIR as well as the delineation of the subendocardial border. Dark blood PSIR also improves the detection of LGE in papillary muscles and thin wall structures such as atria and valves. In several cases, the circumferential extent of the scar appears underestimated by bright blood LGE since a portion of the MI had poor contrast with the blood pool. It is also challenging to accurately measure the transmurality of subendocardial MI using bright blood LGE in cases where the subendocardial border is cannot be delineated.

Atrial walls and valves are thin walled fibrous structures with higher collagen fraction resulting in higher gadolinium concentration and shorter T1, which thereby null at shorter TI. These structures may be difficult to resolve from the adjacent blood pool using bright blood LGE but are more readily detected using the dark blood approach (Fig. 9). In order to detect atrial fibrosis, it may be necessary to adjust the displayed window and level or to acquire images with the T1 value adjusted to a lower value than the myocardium in order to null the atrial wall for better visualization of atrial scar. This is an area of future investigation.

Dark blood PSIR LGE is typically reconstructed with a negative blood pool signal which, after windowing, appears darker than the remote normal myocardium. The normal remote myocardium is acquired at or close to the null point and should be near zero. Since the displayed image is always positive, contrast between the normal myocardium and blood is achieved by setting the display window width and level to bias the myocardium to be slightly positive. The bias should be kept small such that the apparent contrast enhancement ration (CER) between the MI and remote normal myocardium is kept adequately high.

### CNR Measurement

**Fig. 10:**
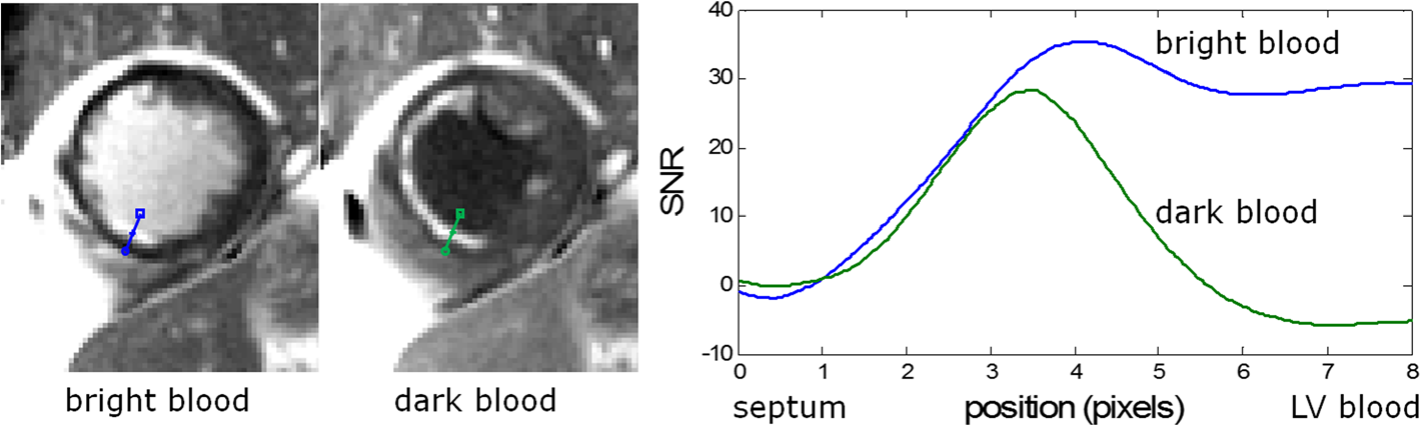
Profiles of SNR vs position thru subendocardial MI for bright blood (*blue*) and dark blood (*green*) PSIR LGE protocols

The CNR_MI-remote_ for the dark blood protocol was slightly less than for the bright blood protocol as expected due to the T2 weighting. The expected loss is approximately exp (−TE/T2)* $\sqrt{2}$ since the dark blood protocol averaged double the measurements (16 vs 8). The TE is picked by the sequence to achieve the desired blood suppression. The measured loss of 13 % is consistent with a TE between 20–25 ms. It is difficult to delineate the subendocardial border for bright blood LGE as illustrated in Fig. 10. Signal intensity profiles thru the MI and blood (Fig. 10) are in SNR units and the subendocardial MI is 1–2 pixels width. It is possible to measure the width of the MI using full-width half maximum criteria for mitigating partial volume effects using the DB PSIR LGE, however this is not possible for the bright blood image.

### Limitations

The proposed dark blood PSIR LGE method uses an IR-T2 preparation and has a minimum delay time due to the duration of the T2 prep. Therefore, for high contrast doses that result in very short T1 it may not be possible to achieve a short enough delay. In these cases, the degree of blood suppression specified may not be achievable. The sequence will attempt to reach the desired blood suppression specified by the delta parameter and if that is not possible will then try to reduce the blood suppression until a solution is achieved.

The loss in CNR is compensated for by increasing the averaging. Roughly double the time is required to achieve the same performance as the bright blood PSIR MOCO. For a short axis stack of 9-slices, this acquisition time is roughly 5 min for dark blood vs 2.5 min for bright blood. This imposes a limitation on the clinical workflow, although it is comparable to some breath-held acquisitions. The user interface for the dark blood PSIR requires a T1 mapping scout and entry of T1 values from roughly measured ROIs in the myocardium and LV blood pool. This process is fairly quick (<1 min) but is an added level of complexity in the clinical workflow.

The display of dark blood PSIR images requires window levelling the PSIR image so that the blood is darker than the normal myocardium. This equates to making the normal myocardium slightly gray (elevated) even though the myocardium may in fact be nulled. Detection of subtle LGE may require careful adjustment of the window level subtle and experience to gain confidence in interpretation.

In this study, the T1 value of blood measured in the MOLLI scout used for setting the protocol is based on a ROI drawn in the left ventricular (LV) cavity. Since the deoxygenated blood of the right ventricular (RV) cavity has T1 that is shorter than the LV blood (typically 150 vs 250 ms), the RV blood pool will have a slight attenuation by the T2 prep and will null slightly earlier. As a result, the RV blood pool suppression will be less than the LV blood. In general, the RV blood was suppressed to provide adequately contrast, and it is possible to further reduce the RV blood signal by increasing the suppression parameter.

The measurement of bright and dark blood PSIR LGE were performed within 1 min, however the order of sampling was not randomized in this study. The contrast does not change significantly during 1 min so it felt that any bias in measurement of contrast would not alter the overall conclusion.

## Conclusions

Free-breathing, dark blood PSIR LGE imaging has been demonstrated to improve the visualization of subendocardial MI and fibrosis in cases with low contrast with adjacent blood pool. The proposed method also improves visualization of thin walled fibrous structures such as atrial walls and valves, as well as papillary muscles. Imaging parameters are calculated automatically based on a single scout scan.

## Abbreviations

CNR: Contrast-to-noise ratio
DB: Dark blood
IR: Inversion recovery
LGE: Late gadolinium enhancement
LUT: Look-up table
MOCO: Motion correction
MOLLI: MOdified Look locker inversion recovery
MT: Magnetization transfer
PSIR: Phase sensitive inversion recovery
VT: Ventricular tachycardia
